# Mobilizing endogenous neuroprotection: the mechanism of the protective effect of acupuncture on the brain after stroke

**DOI:** 10.3389/fnins.2024.1181670

**Published:** 2024-04-25

**Authors:** Tian-cong Fu, Guan-ran Wang, Yu-xuan Li, Zhi-fang Xu, Can Wang, Run-chen Zhang, Qing-tao Ma, Ya-jing Ma, Yi Guo, Xiao-yu Dai, Yang Guo

**Affiliations:** ^1^Tianjin Key Laboratory of Acupuncture and Moxibustion, First Teaching Hospital of Tianjin University of Traditional Chinese Medicine, Tianjin, China; ^2^National Clinical Research Center for Chinese Medicine Acupuncture and Moxibustion, Tianjin, China; ^3^Research Center of Experimental Acupuncture Science, Tianjin University of Traditional Chinese Medicine, Tianjin, China

**Keywords:** ischemic stroke, endogenous brain neuroprotection, acupuncture, astrocytes, microglia, brain blood vessels

## Abstract

Given its high morbidity, disability, and mortality rates, ischemic stroke (IS) is a severe disease posing a substantial public health threat. Although early thrombolytic therapy is effective in IS treatment, the limited time frame for its administration presents a formidable challenge. Upon occurrence, IS triggers an ischemic cascade response, inducing the brain to generate endogenous protective mechanisms against excitotoxicity and inflammation, among other pathological processes. Stroke patients often experience limited recovery stages. As a result, activating their innate self-protective capacity [endogenous brain protection (EBP)] is essential for neurological function recovery. Acupuncture has exhibited clinical efficacy in cerebral ischemic stroke (CIS) treatment by promoting the human body's self-preservation and “Zheng Qi” (a term in traditional Chinese medicine (TCM) describing positive capabilities such as self-immunity, self-recovery, and disease prevention). According to research, acupuncture can modulate astrocyte activity, decrease oxidative stress (OS), and protect neurons by inhibiting excitotoxicity, inflammation, and apoptosis via activating endogenous protective mechanisms within the brain. Furthermore, acupuncture was found to modulate microglia transformation, thereby reducing inflammation and autoimmune responses, as well as promoting blood flow restoration by regulating the vasculature or the blood–brain barrier (BBB). However, the precise mechanism underlying these processes remains unclear. Consequently, this review aims to shed light on the potential acupuncture-induced endogenous neuroprotective mechanisms by critically examining experimental evidence on the preventive and therapeutic effects exerted by acupuncture on CIS. This review offers a theoretical foundation for acupuncture-based stroke treatment.

## 1 Background

Ischemic stroke (IS) remains the second leading cause of death and disability in adults worldwide. According to research (Wang, 2005; Neuhaus et al., 2017; Ornello et al., 2018), the combination of thrombolytic and neuroprotective agents is the first-line treatment for acute ischemic stroke (AIS). However, commonly used thrombolytic drugs, such as the recombinant tissue plasminogen activator (rtPA), have several drawbacks, including a narrow effective time window (<4.5 h) and bleeding risk, among other disadvantages. Furthermore, only ~5% of patients finally receive rtPA, with the vast majority only receiving supportive care in the acute AIS phase (Montaño et al., 2013; Chang et al., 2015; Auboire et al., 2021). Although more than 1,000 neuroprotective agents could ameliorate nerve damage post-AIS over the past 50 years, most of them failed in large-scale clinical trials (Park et al., 2012; Kim et al., 2017). Despite the current experimental studies on stroke treatment primarily focus on protecting neurons from ischemic pathogenic factors, such as excitatory neurotransmitter toxicity, oxidative stress (OS), inflammation, and apoptosis, applying these findings to clinical practice poses a significant challenge. In this context, a recent study has added a vital perspective, reporting that the brain possesses inherent self-protection mechanisms, which can fully mobilize endogenous protective measures, and harnessing this natural defense mechanism is identified as crucial for stroke prevention and treatment (Iadecola and Anrather, 2011). Endogenous brain protection (EBP) encompasses a collaborative process of multicellular programs; if one cell type is threatened, a coordinated multicellular response emerges to maintain the tissue's dynamic equilibrium, resulting in a coherent defense system program that can prioritize the survival of critical cells. By acting on the coordinated neuro–glial–vascular protection process, EBP induces multicellular cooperation between neurons, astrocytes, microglia, T cells, and cerebral vessels. In order to maintain internal tissue stability when a specific cell type is threatened, this process might be initiated to confront OS, excitatory toxicity, inflammation, apoptosis, and other ischemic cascade reactions, thereby reducing ischemic injury and prioritizing the survival of critical cells (Datta et al., 2020). Therefore, the prospective promotion of stroke research is primarily based on comprehensively strengthening endogenous protective measures.

In the history of Chinese medicine, acupuncture has been used to treat diseases for thousands of years. Its functions include dredging meridians, harmonizing yin and yang, and boosting positive qi. In *Ling Shu, Nine Needles and Twelve Originals (*灵枢·九针十二原*)*, acupuncture is described as “Using microneedles to unblock the meridians, harmonize qi and blood, and improve the in and out flow of qi and blood.” Homeostasis regulation, which enhances the body's endogenous protection to attain disease prevention and treatment goals, is the fundamental mechanism of acupuncture (Zhang et al., 2020; Jia et al., 2022).

High-quality clinical evidence-based theory has confirmed the medical efficacy and safety of acupuncture in treating acute ischemia (MacPherson et al., 2013; Qiu, 2013; Liu et al., 2015, 2016a; Xin et al., 2015; Zhao et al., 2017; Zhan et al., 2018). Specifically, recent studies have shown that acupuncture exerts a synergistic effect on EBP cells (astrocytes, microglia, and vascular cells, among others) (Cao et al., 2021). Therefore, to elucidate the theoretical evidence for ischemic stroke prevention and treatment, this review summarizes the mechanism of acupuncture in mobilizing endogenous neuroprotection.

## 2 Stroke and its endogenous protection mechanism

After stroke, the supply of oxygen and glucose to the brain is impeded, resulting in an inadequate production of ATP to meet the brain's energy demands. Consequently, brain cells die quickly due to energy loss, which is particularly severe in areas with the lowest blood flow. Furthermore, in the relatively mild ischemic area (ischemic penumbra), a continuous depolarization wave induces the release of neurotransmitters, accompanied by a glial cell reuptake disorder, causing extracellular glutamate and other excitatory neurotransmitters to aggregate and produce excitatory toxicity. Continuous glutamate receptor activation leads to the accumulation of extracellular calcium ions, eventually resulting in a chain of ischemic cascade reactions, including antioxidant stress, excitotoxicity, and inflammatory apoptosis. Meanwhile, ischemia also triggers a cascade of inflammatory signals, causing white blood cell aggregation in blood vessels and between cells. By producing cytotoxic mediators, these inflammatory cells further damage the brain tissue, eventually causing brain damage.

To alleviate brain injury and restore internal environment homeostasis, the brain's local and remote protection mechanisms can resist harmful events as follows.

Early IS will result in reactive astrocyte production, cell proliferation and rapid aggregation, and glial scar formation around the lesions (Sun et al., 2019; Tanabe et al., 2019; Kim et al., 2021), thereby inhibiting or aggravating neuronal injury. On the one hand, activated astrocytes can protect neurons through the inhibition of glutamate excitatory toxicity by antioxidant stress and anti-inflammatory responses, among other neuroprotective effects (de Pablo et al., 2013; Jeong et al., 2014; Roy Choudhury et al., 2014); on the other hand, the glial scar could increase inflammation and intracerebral pressure and decrease vascular perfusion and the accelerated brain edema process (Kim et al., 2015).

Microglia, a permanent immune cell in the brain, is involved in the first-line central nervous system (CNS) innate immunity defending the brain against injury and diseases (Franco-Bocanegra et al., 2019). Following ischemic brain injury, microglia rapidly migrate to the lesion site, eliminate cell debris, and generate anti-inflammatory/pro-inflammatory cytokines (Ma et al., 2017). Microglia activation post-IS was previously assumed to aggravate brain injury. However, at present, growing evidence indicates that microglia activation may reduce neuronal apoptosis and exert beneficial effects on later neural repair stages (Sherafat et al., 2021).

Increasing arterial pressure by activating the sympathetic nerve-releasing hormone during cerebral ischemia enhances blood flow through communicating branch vessels (collateral circulation) adjacent to the normal perfusion area, aiding blood supply to the ischemic area. Within 24 h post-stroke, T cells appear in the brain tissue and could infiltrate the brain for a long time (Gelderblom et al., 2009), exerting an essential impact on stroke outcomes.

The clearing function of the “brain lymphatic system” has neuroprotective effects (Toro et al., 2019; Cheng and Wang, 2020; Segawa et al., 2021). Some anti-inflammatory and neuroprotective cytokines produced by regulatory lymphocytes, including interleukin 10 (IL-10) and transforming growth factor β (TGF- β), can limit white blood cell infiltration and suppress natural and acquired immune responses, thus promoting the survival of ischemic neurons. The protective signaling pathway activated in the late stages of ischemic cascade reactions can promote the repair of injured brain tissues. Furthermore, growth factors secreted by microglia, macrophages, neurons, astrocytes, and intravascular cells, such as erythropoietin (EPO) and insulin-like growth factor 1 (IGF-1), can be produced by peripheral organs and infiltrate the brain through cerebral vessels (Jelkmann, 2005). Meanwhile, following ischemia, neural precursor cells and bone marrow-derived endothelial progenitor cells infiltrate the ischemic injury site, restoring tissue homeostasis and reconstructing neural networks by recombining the extracellular matrix (ECM) and replacing damaged cells, thus playing a crucial role in brain microvascular network reconstruction.

## 3 Acupuncture plays a neuroprotective role by regulating astrocytes

Astrocytes are positioned between the cell body and nerve cell processes. They function by supporting and guiding neurons and enhancing their survival. Notably, astrocytes are the brain's most widely distributed cell type. The location, size, and time of the physical barrier formed by reactive astrocytes influence the acceleration of nerve cell death. Furthermore, research shows that acupuncture can play the role of an EBP through astrocyte regulation.

### 3.1 Acupuncture can reduce oxidative stress and inhibit excitatory toxicity by regulating astrocytes after stroke

Acupuncture could impede the sudden depletion of oxygen after excitotoxicity in IS, and when the endogenous redox balance cannot be maintained, OS will occur, yielding reactive oxygen species (ROS) and activating the release of various inflammatory factors (Sun et al., 2008; Shen et al., 2012; Jiang et al., 2013; Lin et al., 2022), as well as destroying the integrity of the blood–brain barrier (BBB) and increasing the infarction volume. The nuclear factor E2-related factor 2 (Nrf2), which plays a vital role in cell redox homeostasis, is expressed in astrocytes (Yang et al., 2016; Hoxhaj and Manning, 2020; Terada et al., 2020). According to research (Jin, 2017), electroacupuncture (EA) can regulate downstream targets along the Nrf2 signaling pathway γ- (Choudhury and Ding, 2015). Additionally, GCS expression can enhance the body's antioxidant capacity and influence ischemic cerebrovascular diseases. Zhao showed that acupuncture at “Baihui” and “Sishencong” can significantly upregulate antioxidant enzymes SOD and GSH-px in the ischemic penumbra of middle cerebral ischemia reperfusion (MCAO/R) rats, with its mechanism of action speculated to be associated with the activation of the Nrf2 signaling pathway (Zhao and Ma, 2018).

**Figure 1:**
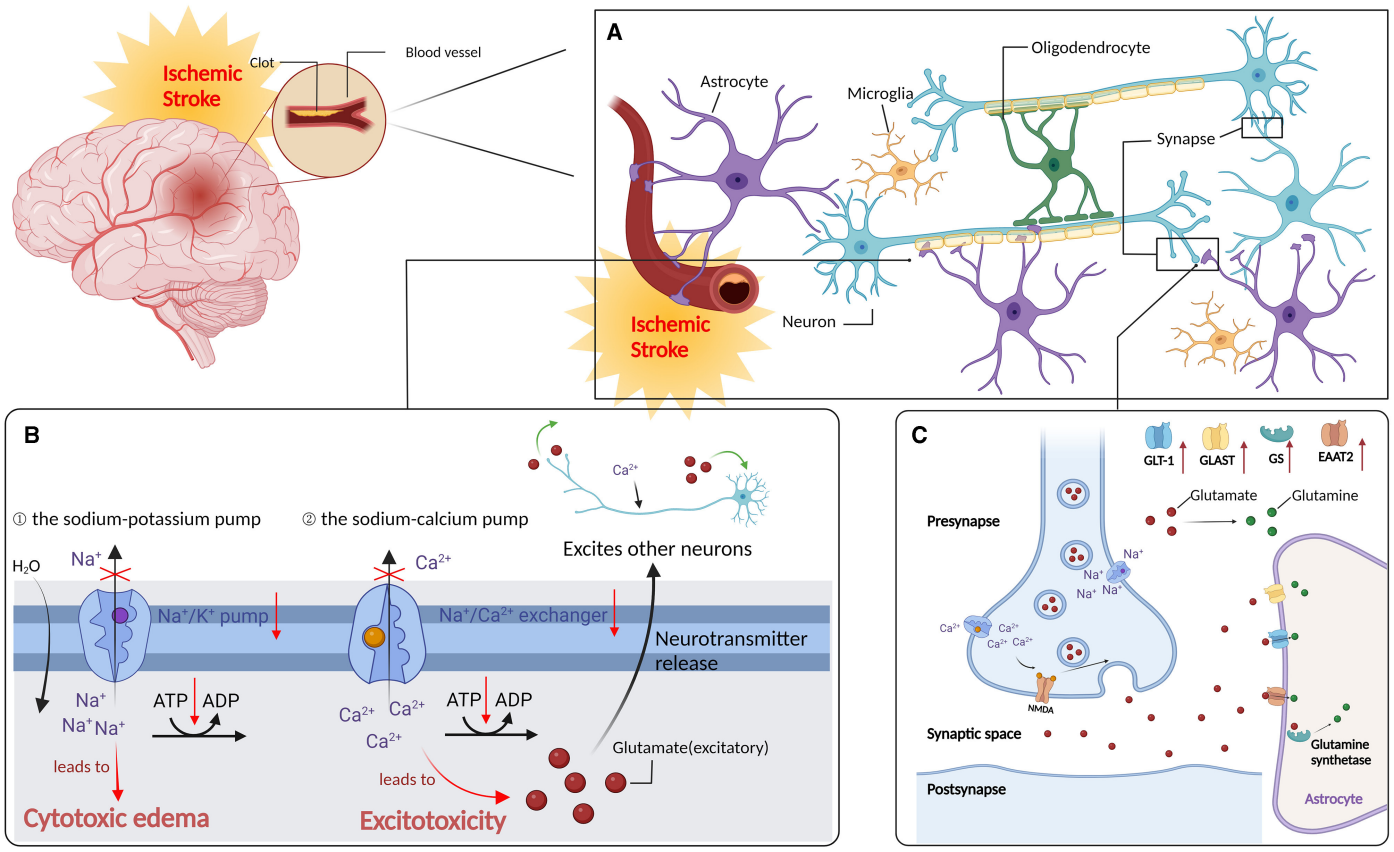
Regulate astrocytes to reduce oxidative stress and after stroke. **(A)** Ischemic stroke: distribution of neurone in the brain. **(B)** Ischemic cascade: after an ischemic stroke, neurons in the ischemic core are rapidly deprived of oxygen due to lack of blood supply, resulting in reduced ATP production, which in turn lead to the failure of Na^+^/k^+^ pumps (1) and plasma membrane Ca2+/ATP pumps (2). **(C)** Acupuncture can enhance the ability of astrocytes to clear glutamate, inhibit the expression of glutamate NMDA receptor NR1, NR2B and other proteins, up-regulate the expression of glutamate transporter-1 (GLT-1), NR2A, cannabinoid receptor type 1 and type 2 proteins; expression of glutamate asparate transporter (GLAST) and glutamine synthetase (GS), and reduce the MCAO rat brain tissue glutamate content and attenuate neurotoxicity, thus exerting a protective effecr in ischemic brain tissues. In addition, electropuncture pretreatment could reduce glutamate toxicity by upregulating EAAT2 and activating CB1R in the ischemic penumbra of AS, thus protecting neurons from cerebral ischemia (the red upward arrow indicates that acupuncture promotes its upregulation).

Upon neuronal anoxic depolarization, Ca2+ flows into the terminals of presynaptic neurons, resulting in an abundance of excitatory neurotransmitter glutamate released into the synaptic space, thereby causing excitotoxicity. Depending on their specific Na+-dependent glutamate transporter, astrocytes absorb glutamate [glutamate aspartate transporter (GLAST) and glutamate transporter-1 (GLT-1)] from the extracellular space, regulate the extracellular Ca2+ levels, and critically influence injured neuron repair (Kobayashi et al., 2019; Pajarillo et al., 2019). Acupuncture can enhance the astrocyte glutamate clearance ability; inhibit the expression of glutamate NMDA receptors (NR1 and NR2B) and other proteins; and upregulate GLT-1, NR2A, and cannabinoid receptor type 1 (CB1R) and type 2 (CB2R) proteins, by reducing neurotoxicity, thus protecting the ischemic brain tissue (Dai, 2016). Furthermore, acupuncture at “Neiguan” can upregulate the GLAST and glutamine synthetase (GS) and downregulate glutamate in the brain tissue of MCAO rats. Furthermore, acupuncture at “Neiguan” will increase the astrocyte GLT1 and GS protein expression, reduce excessive glutamate, and upregulate glutamine synthetase in the brain tissue of MCAO rats. As the main transporter for clearing excitatory neurotransmitter glutamate from the CNS, the excitatory amino-acid transporter 2 (EAAT2) is responsible for the reuptake of more than 90% of glutamate in the brain and is abundant in astrocytes (Danbolt, 2001; Wei et al., 2019). Electroacupuncture pretreatment can minimize glutamate toxicity by upregulating EAAT2 and activating astrocyte CB1R in the ischemic penumbra, thereby protecting neurons from cerebral ischemia. Furthermore, the GS-mediated glutamine synthesis helps astrocytes restore extracellular glutamate to normal levels and protect neurons from ischemia/reperfusion (I/R) injury (Stelmashook et al., 2011) (Figure 1).

### 3.2 Acupuncture regulates astrocytes to reduce inflammatory reactions

Astrocytes are critically involved in the brain's inflammatory network. After IS, pro-inflammatory factors rapidly induce pathophysiological changes in astrocytes, including reactive astrocyte hypertrophy and proliferation, vimentin and glial fibrillary acidic protein (GFAP) overexpression, and cytokine and chemokine production (IL-6, TNF- α, IL-1 α and β, IFN-γ, etc). Between 4 and 24 h post-IS, a multitude of astrocytes and glial scars appeared in the lesion's core area, peaking around the fourth day (Choudhury and Ding, 2015). From the neurotoxicity and neurotrophic perspectives, the glial scar has two characteristics. First, cytokines released from the glial scar can directly or indirectly induce neurotoxicity media [such as nitric oxide (NO)] and increase BBB permeability, thereby inducing neuronal death, promoting further cerebral infarction development, preventing inward axonal growth, and affecting nerve regeneration during recovery post-IS. Second, the scar can be used as a barrier to isolate damaged tissues from healthy tissues, preventing additional damage to surrounding tissues.

Acupuncture can upregulate GFAP in a cerebral ischemia astrocyte model and promote astrocyte activation (Tao et al., 2016). Paulina et al. found that EA preconditioning can downregulate the I/R injury-induced N-myc downstream-regulated gene 2 (NDRG2) (Vaitkiene et al., 2017), reduce stroke-induced astrocyte apoptosis, and exert neuroprotective effects. After 24 h of modeling, MCAO rats were treated with scalp acupuncture in combination with exercise therapy. According to the results, BDNF and GFAP were upregulated in the cortex and around the rats' striatum, which could have inhibited the inflammatory reaction and alleviated ischemic brain injury. Furthermore, Tao et al. found that treating MCAO rats with EA on the third day after modeling could promote the proliferation of GFAP+/vimentin+/nestin+reactive astrocytes, upregulate BDNF, and exert a neuroprotective effect. Cheng et al. (2014a,b) demonstrated that the combination of EA and induced pluripotent stem cell-derived extracellular vesicles could regulate the IL-33/ST2 axis and inhibit the activation of microglia and astrocytes 72 h after stroke, thereby exerting a neuroprotective effect.

## 4 Acupuncture can enhance the neuroprotective effect of microglia

As an intrinsic brain immune cell, microglia serve as the primary barrier against injury to the CNS (Colonna and Butovsky, 2017). Following ischemic brain injury, microglia rapidly migrate to the lesion site, eliminate cell debris, and produce anti-inflammatory/pro-inflammatory cytokines. It was previously assumed that microglia activation post-IS might aggravate brain injury. However, at present, growing evidence shows that microglia activation may reduce neuronal apoptosis and benefit the later nerve repair stages (Tian and Mao, 2022). Under normal physiological conditions, microglia exhibit a small cell body or branch shape and have a monitoring function (Bao et al., 2018; Souder et al., 2021). This condition is referred to as the “static microglia” state and disrupting brain homeostasis can induce microglia activation.

### 4.1 Acupuncture can downregulate inflammatory factors by modulating microglia

When activated by ischemia, microglia rapidly shift from a static to an active state. This transition is known as microglia polarization. Polarization is categorized into M1 and M2 phenotypes. On the one hand, M1 microglia secrete various inflammatory cytokines (including IL-1 β, IL-6, TNF-α, IFN-γ, and INOS) (Collmann et al., 2019) and neurotoxic mediators (MMP9 and MMP3), which cause BBB destruction and ECM degradation, resulting in nerve degeneration or death. The activation phenotypes in this process are CD11b (Liu et al., 2018), CD16 (Jiang et al., 2018), CD32 (Jin et al., 2014), and CD86 (Li et al., 2018). On the other hand, M2 microglia are reparative and can secrete anti-inflammatory factors (IL-10, IL-4, IL-13, IGF-1, and TGF-β), as well as the insulin-like growth factor (IGF) and the vascular endothelial growth factor (VEGF), to inhibit inflammation and promote angiogenesis and tissue repair (Zhu et al., 2019), with CD206 as the activated phenotype (Shu et al., 2016). Therefore, the M1/M2 change is the embodiment of microglia's dual characteristics in inflammatory responses, through which it critically influences stroke prognosis.

As a vital microglia-related inflammatory signaling pathway, NF-κ B can effectively induce inflammatory cytokine (TNF-α, IL-1 β, IL-6, etc.), chemokine (monocyte chemoattractant protein-1), adhesion molecule (ICAM-1 and VCAM-1), and other expressions to aggravate the inflammatory cascade reaction (Li et al., 2019; Meng et al., 2019; Xu et al., 2019). Some studies have shown that (Liu et al., 2002, 2016b) acupuncture can regulate the microglia activation direction and that EA can downregulate M1 microglia markers (Iba-1 and CD11b) in the ischemic focus of the cerebral ischemia model. They also found that inhibiting the NF-κ nuclear translocation of B p65 suppresses p38 mitogen-activated protein kinase (p38 MAPK) and myeloid differentiation factor 88 (MyD88) expression in the sensorimotor cortex around the infarction region and downregulates TNF-α, IL-1 β, and IL-6, thereby reducing the transformation from microglia to the M1 phenotype. Han et al. (2015) demonstrated that acupuncture at “Neiguan” and “Quchi” can suppress microglia activation and inhibit microglia involvement in the TLR4/NF-κ signaling pathway. Acupuncture-induced abnormal expression of the B signaling pathway factor downregulates TNF-α, IL-1 β, and IL-6, reducing the neurological function score of the MCAO model and necrosis of hippocampal neurons in the ischemic focus. In a bilateral carotid ischemia model, acupuncture can upregulate the nuclear translocation of Nrf2 in neurons and downstream target genes [NADPH quinone oxidoreductase 1 (NQO1) and heme oxygenase 1(HO1)] by reducing the activation of Nrf2-dependent microglia, which exerts a neuroprotective effect and improves bilateral carotid ischemia-induced cognitive impairment (Wang et al., 2015). Table A1 details the effect of acupuncture on microglia polarization post-IS.

## 5 Acupuncture can regulate homeostasis by enhancing cerebral blood flow or BBB and exert a neuroprotective effect

IS is often followed by brain network dysfunction, resulting in sudden nerve function defects. Therefore, to save the neurovascular unit, it is essential to restore blood flow perfusion. Blood flow recovery post-IS can be achieved through thrombolysis or mechanical recanalization. However, in some patients, reperfusion may aggravate the initial ischemia-induced injury, resulting in the so-called “brain reperfusion injury.” According to research, acupuncture can maintain the integrity of the BBB, expand blood vessels, accelerate blood flow, enhance microcirculation, increase cerebral blood flow (CBF), relieve cerebral ischemia and hypoxia, promote cerebral collateral circulation (CCC), and reduce brain tissue damage.

### 5.1 Acupuncture can maintain the integrity of the BBB

The BBB is a multicellular vascular structure between the CNS and the peripheral blood circulation, which can limit the entry of pathogens, blood solutes, and macromolecules or hydrophilic molecules into the cerebrospinal fluid while maintaining the stability of the brain environment. Due to the distinct pathogenesis of IS, I/R injury causes BBB destruction and increases vascular permeability and brain edema, resulting in secondary brain injury (Moskowitz et al., 2010; Feng et al., 2022). Therefore, maintaining BBB integrity is one of the main objectives of brain protection post-IS.

Wu et al. (2001) found that EA can limit the area of Evans blue extravasation and reduce BBB damage post-I/R, implying that EA can regulate brain homeostasis after stroke by protecting BBB integrity and inhibiting NOX4 and ROS production. Xu et al. (2018) found that EA can downregulate AQP4 in the striatum of MCAO/R rats (AQP4 is the most abundant aquaporin in brain tissue and is expressed on the terminal foot around the BBB vessels wrapped by astrocytes), reduce brain edema and BBB damage, and exert a brain-protective effect. Additionally, Shen et al. (2009) showed that EA preconditioning can alleviate cerebral ischemia-induced brain edema and BBB dysfunction by downregulating matrix metalloproteinase-9 (MMP-9). Lin et al. also observed that EA treatment can inhibit MMP-2/MMP-9 expression in rats with cerebral ischemia, thus promoting brain protection. In this regard, because of its ability to maintain BBB integrity, acupuncture has become one of the key EBP measures.

Increasing oxygen and blood supply to brain tissue after stroke is crucial for preventing and treating brain damage. EA stimulation of “Zusanli” has been shown to increase CBF in rats with cerebral ischemia (Hsieh et al., 2006). Similarly, stimulating “Shuigou” with EA can also promote vascular endothelial cell proliferation and increase the local CBF of rats (Du et al., 2011). Additionally, stimulating “Fengfu” and “Shendao” with EA can significantly reduce ischemia-induced glutamate release and transient CBF increase during reperfusion to protect neurons from I/R injury, thereby exerting a neuroprotective effect (Pang et al., 2003). In conclusion, acupuncture is an exogenous therapeutic strategy that can improve microcirculation, regulate cerebral vascular reserve or increase CBF, mobilize brain tissue energy metabolism to reduce brain injury, and enhance the endogenous protective effect.

### 5.2 Acupuncture can regulate cerebrovascular reserve

Cerebrovascular reserve (CVR) is the ability to adjust the CBF stability to meet the demands of brain parenchymal metabolism via compensatory relaxation and contraction of intracranial arterioles and capillaries under physiological or pathological stimulation, which is the key mechanism of homeostasis regulation post-IS. Some domestic studies (Zhang and Tian, 2020) showed significantly improved clinical symptoms in acute cerebral infarction patients after intravenous thrombolysis by the “awakening the brain and opening the body” acupuncture method compared to the control group without acupuncture treatment and that CVR index improvement was found to be better in the treatment group than in the control group through transcranial Doppler ultrasound detection. Moreover, it is suggested that the “Xingnao Kaiqiao” acupuncture method can adjust CBF to realize its brain protection effect.

### 5.3 Acupuncture can enhance cerebral collateral circulation

As one of the endogenous compensatory mechanisms in the brain, collateral circulation (CC) is one of the important determinants of IS outcomes. However, within 3–5 days post-IS, brain edema continues to deepen, and CC establishment is also affected by the expansion of the infarction region (Campbell et al., 2013).

Shi et al. (2017) discovered that the number of blood vessels was significantly reduced around the infarction area in the MCAO group at the first, third, and sixth hours after replicating the MCAO model. Compared to the MCAO group, the number of blood vessels in the EA group was relatively higher, implying that EA intervention improved the CC around the infarction area, resulting in a corresponding increase in CBF. According to Sun et al. (2020) and other clinical studies, acupuncture intervention can expand cerebral blood vessels, increase CBF, reduce the infarction area, promote CCC formation, and improve motor function. The speculated mechanism underlying this process is that after EA stimulates the body's reflex center, bioelectric effects are transmitted to the cerebral cortex through nerves, changing the excitability of neurons in the cerebral cortex and accelerating CCC formation. Additionally, Qi et al. showed that acupuncture can improve the ischemia and hypoxia status of the ischemic focus, as well as promote CC formation and hematoma absorption. Therefore, after IS, acupuncture can promote the establishment of the vascular CCC, increase CBF, and improve the body's EBP.

## 6 Acupuncture mediates endogenous neuronal protection by enhancing T cell

Following a hemorrhagic stroke, the immune system adapts to the ischemic state to protect against post-stroke damage to the central nervous system. Once the blood–brain barrier is broken, immune cells including pro-inflammatory factors in the peripheral blood circulation can invade the central nervous system. Moreover, inflammatory cells [neutrophils (Cai et al., 2020), monocytes, macrophages, various types of T cells, and other inflammatory cells] in the peripheral blood can cross the blood–brain barrier to the ischemic area where they cause a systemic inflammatory response (Gan et al., 2014; Perez-de-Puig et al., 2015), thereby increasing the risk of secondary infarction and delaying neurological recovery (Magnus et al., 2012). Therefore, a strategy should be developed for reducing the damage caused by inflammatory cytokines after stroke. Within 24 h after the occurrence of ischemic stroke, T cells are released in the brain tissue where they infiltrate the tissues for a long time. Research has shown that compared with healthy individuals, stroke patients show increased expression of HLA-DR and CD25 on T cells, and the surviving T cells in peripheral blood are activated and secrete pro-inflammatory cytokines (Gill and Veltkamp, 2016). In an experimental mouse model of cerebral ischemic stroke, lymphocyte loss may occur which reduces the number of CD4+T cells, resulting in cell apoptosis, while the delayed recovery of CD4+T cell count indicates an increase in the risk of subsequent infection (Prass et al., 2003). Studies have shown that acupuncture can reduce the infiltration of inflammatory cells, downregulate the expression of CD4+in cells, improve nerve function scores, reduce the expression of Th17 cells (Lee et al., 2016), and inhibit the secretion of mouse pro-inflammatory cytokine IL-17 (Liu et al., 2013). This suggests that CD4+T cell count may be closely related to the degree of multiple immunosuppression induced by cerebral ischemia, that is, stroke is accompanied by T-cell activation, and this can be prevented by acupuncture treatment.

Regulatory T cells (Tregs) have been shown to inhibit excessive immune response and maintain immune tolerance and homeostasis. Tregs cells can prevent the expansion of secondary infarction by alleviating excessive production of pro-inflammatory cytokines and regulating the invasion and/or activation of lymphocytes and microglia in the ischemic brain. A study by Chamorro et al. (2012) showed that Tregs may exert an anti-inflammatory role by secreting the anti-inflammatory cytokine IL-10 and can cross the blood–brain barrier, and IL-10 gene knockout will promote an increase in infarction (Liesz et al., 2009). Serra et al. (2003) reported that Tregs inhibit inflammation by secreting the anti-inflammatory cytokine IL-10. IL-10 gene knockout will lead to an infarction increase (Liesz et al., 2009). Tregs can promote the expression of dendritic cells (DCs) and reduce the activation of DCs on effector T cells 9, which damages the blood–brain barrier and promotes leukocyte infiltration and brain injury. Within 24 h of cerebral ischemia, Treg cells demonstrate the capacity to suppress MMP-9 production. Moreover, they exert a protective effect against ischemic stroke by suppressing the expression of metalloproteinase-9 (MMP-9) (Li et al., 2013). However, when they are cultured in the extracellular pore, Tregs may lose their inhibitory effect on MMP-9. This indicates that Tregs can exert this inhibitory effect by interacting with neutrophils. Xu et al. (2014) showed that acupuncture or electroacupuncture stimulation of “Baihui” (GV20) and “Zusanli” (ST36) can significantly reduce inflammatory cell infiltration and the expression of pro-inflammatory metalloproteinase-2 (MMP-2) in rats with ischemia–reperfusion injury. It also significantly downregulates the expression of aquaporins AQP4 and AQP9 in the ischemic brain, thereby reducing inflammation and brain edema, demonstrating its brain-protective effect.

## 7 Acupuncture plays a neuroprotective role by enhancing the clearing function of the “brain lymphatic system”

The glymphatic system (GS) is a newly discovered system that clears waste from the central nervous system. Studies have found (Rasmussen et al., 2018; Chen et al., 2021; Lv et al., 2021; Ren et al., 2021; Zhou et al., 2021) that the “glymphatic system (GS) of the brain” closely regulates inflammation-related pathways and maintains the balance of clearing and accumulating harmful metabolic substances (such as Aβ and Tau) in the brain. This system facilitates the dynamic flow and exchange of materials between the cerebrospinal fluid (CSF) and interstitial fluid (ISF) through the polar distribution of aquaporin-4 (AQP4) on astrocytes. The GS is functionally connected to meningeal lymphatics (ML), CSF, and the BBB, collectively participating in the metabolic clearance process of Aβ, Tau, and other waste proteins. Imbalances in its functioning are implicated in stroke, Alzheimer's disease, and other neurological disorders. Moreover, recent studies have highlighted the role of the GS in melatonin-mediated neuroprotection and neuroinflammation characterized by pyroptosis (Li et al., 2020; Chen et al., 2021; Lv et al., 2021; Lyu et al., 2021a,b; Zhou et al., 2021). A recent study (Zhong et al., 2022) discovered that electroacupuncture at “Baihui and Shenting” can reduce cell pyroptosis by modulating the expression level of endogenous melatonin, thereby inhibiting neuroinflammation and activating plasma cells in the CA1 area of the hippocampus. This treatment approach also mitigates neurological and cognitive impairments in rats with cerebral ischemia–reperfusion injuries (Lin et al., 2015, 2016a,b, 2017).

Given the crucial role of astrocytes in the regulatory mechanism of the brain lymphoid system, along with the latest research advancements in melatonin, inflammatory factors, and other related regulatory pathways in the regulation of Aβ and Tau protein pathological metabolism, it is reasonable to hypothesize that electroacupuncture may have a neuroprotective effect in this process. However, it is important to note that there is currently a lack of experimental research to confirm its effectiveness.

## 8 Summary and outlook

Ischemic stroke is a multistep condition caused by the blockage of blood vessels in the brain. When the brain is deprived of oxygen for more than 60–90 s, the cells in the brain stop working, and irreversible damage occurs over a period of several hours, leading to the death of the brain tissue. Acute ischemic stroke triggers a series of reactions such as oxidative stress, excitotoxicity, and inflammation, and these pathological processes may significantly affect subsequent recovery. Therefore, it is important to mobilize endogenous protection against these pathological processes.

Ischemic stroke is caused by decreased blood and oxygen supply, which leads to neuronal energy metabolism disorders, including decreased intracellular ATP content, mitochondrial dysfunction, and oxidative stress. Disturbed energy metabolism triggers the release of large amounts of glutamate and excitation of glutamate receptors, which in turn increases intracellular calcium levels in neurons and causes excitotoxicity (Choi, 1988; Lipton and Rosenberg, 1994; Arundine and Tymianski, 2003); in addition, disrupted energy metabolism causes mitochondrial dysfunction and elevates oxidative stress (Li et al., 2022), which causes the release of large amounts of reactive oxygen radicals. Hypoxia and energy deficiency negatively affect the brain's antioxidant defense system, resulting in decreased activity of key enzymes such as glutathione, superoxide dismutase, and glutathione peroxidase. Consequently, this amplifies the degree of oxidative stress, ultimately exacerbating neuronal damage and cell death. Therefore, this review mainly explores the five aspects of astrocytes, microglia, cerebrovascular and cerebral blood flow changes, T-cell activation or not, as well as the clearing function of the cerebral lymphatic system. We synthesized findings from contemporary research to examine the pathological alterations in astrocytes following ischemic stroke. Based on the collation of relevant studies, it has been observed that acupuncture can potentially act as a neuroprotective agent by regulating astrocytes to reduce oxidative stress, inhibit excitotoxicity, and suppress inflammatory responses. Furthermore, acupuncture can modulate microglial polarization, thus reducing inflammatory reactions. Additionally, acupuncture has been found to maintain the integrity of the blood–brain barrier, improve microcirculation, optimize cerebrovascular reserve, enhance or establish collateral circulation, and increase cerebral blood flow. Moreover, it can inhibit T-cell activation or enhance the brain's lymphatic system clearance function, thereby exerting a protective effect on brain function.

Acupuncture is the most widely used traditional and complementary medicine, used in 113 of 120 countries according to a 2019 World Health Organization report (World Health Organization, 2019). Acupuncture was most commonly recommended for musculoskeletal and connective tissue diseases; neurological disorders; obstetrics, gynecology and women's health; oncology; and gastrointestinal disorders (Dobos et al., 2012; Cho et al., 2014; Birch et al., 2018). Acupuncture works by stimulating the microenvironment of acupoints, including processes that change the anatomical structure of acupoints, regulating the microenvironment of acupoints, modulating cellular functions, and releasing various bioactive substances. Studies (Chen et al., 2018; Ding et al., 2018) have shown that acupuncture can promote the degranulation of local mast cells at acupoints, accompanied by the generation of electrical signals and the secretion of biochemical substances, such as trypsin-like enzymes, 5-hydroxytryptamine (5-HT), substance P (SP), and histamine (HA), which have been shown to mediate the acupuncture effect. After the accumulation of acupoint sensitization effects, acupuncture signals are transmitted to the central nervous system where integration and regulation of the function and activity of the target organ occur. In addition to this, acupoints can sense mechanical stimuli from the outside world, which may be one of the most initial motivating factors for the effect of acupuncture (Berman et al., 2010). Acupuncture is considered to be a minimally invasive mechanical stimulus (Jin et al., 2019), which does not cause fracture or even necrosis of muscle fibers or accumulation of red blood cells, immune cells, and cellular debris in the muscle interstitial space. Some researchers examined the tissue adhering to acupuncture needles upon removal and identified fragments of collagen fibers, fibroblasts, and adipocytes. This provides additional evidence supporting the notion that acupuncture constitutes a minor traumatic stimulus (Kimura et al., 1992). Localized tissue injury will trigger an acute immune response (Eming et al., 2007), driven by infiltration by several cells including leukocyte and mast cells and the release of vasoactive substances such as HA, SP, and adenosine. Accordingly, this review collected and organized the changes of a series of biological indicators (biochemical measurements) after using acupuncture to stimulate different acupoints (as shown in Table A1). The present results provide a reference for further research into the biological mechanism of acupuncture in the management of ischemic stroke.

## Author contributions

T-cF: writing—original draft, review and editing, and figure and table design. G-rW, Y-xL, and CW: data curation. Z-fX and YiG: writing—review and editing. R-cZ, Q-tM, and Y-jM: literature search. X-yD and YaG: writing—review and editing and funding acquisition. All authors contributed to the article and approved the submitted version.

## References

[B1] Arundine M. Tymianski M. (2003). Molecular mechanisms of calcium-dependent neurodegeneration in excitotoxicity. Cell Calc. 34, 325–337. 10.1016/S0143-4160(03)00141-6 12909079

[B2] Auboire L. Fouan D. Grégoire J.-M. Ossant F. Plag C. Escoffre J.-M.. (2021). Acoustic and elastic properties of a blood clot during microbubble-enhanced sonothrombolysis: hardening of the clot with inertial cavitation. Pharmaceutics 13:1566. 10.3390/pharmaceutics13101566 34683859 PMC8537785

[B3] Bao L.-H. Zhang Y.-N. Zhang J.-N. Gu L. Yang H.-M. Huang Y.-Y.. (2018). Urate inhibits microglia activation to protect neurons in an LPS-induced model of Parkinson's disease. J. Neuroinflamm. 15:131. 10.1186/s12974-018-1175-8 29720230 PMC5932803

[B4] Berman B. M. Langevin H. M. Witt C. M. Dubner R. (2010). Acupuncture for chronic low back pain. N. Engl. J. Med. 363, 454–461. 10.1056/NEJMct0806114 20818865

[B5] Birch S. Lee M. S. Alraek T. Kim T. H. (2018). Overview of treatment guidelines and clinical practical guidelines that recommend the use of acupuncture: a bibliometric analysis. J. Altern. Complement. Med. 24, 752–769. 10.1089/acm.2018.0092 29912569

[B6] Cai W. Liu S. Hu M. Huang F. Zhu Q. Qiu W.. (2020). Functional dynamics of neutrophils after ischemic stroke. Transl. Stroke Res. 11, 108–121. 10.1007/s12975-019-00694-y 30847778 PMC6993940

[B7] Campbell B. C. Christensen S. Tress B. M. Churilov L. Desmond P. M. Parsons M. W.. (2013). Failure of collateral blood flow is associated with infarct growth in ischemic stroke. J. Cereb. Blood Flow Metab. 33, 1168–1172. 10.1038/jcbfm.2013.77 23652626 PMC3734777

[B8] Cao B. Tan F. Zhan J. Lai P. (2021). Mechanism underlying treatment of ischemic stroke using acupuncture: transmission and regulation. Neural Regener. Res. 16:944. 10.4103/1673-5374.297061 33229734 PMC8178780

[B9] Chamorro Á. Meisel A. Planas A. M. Urra X. van de Beek D. Veltkamp R.. (2012). The immunology of acute stroke. Nat. Rev. Neurol. 8, 401–410. 10.1038/nrneurol.2012.98 22664787

[B10] Chang D. Wang Y.-C. Bai Y.-Y. Lu C.-Q. Xu T.-T. Zhu L.. (2015). Role of P38 MAPK on MMP Activity in photothrombotic stroke mice as measured using an ultrafast MMP activatable probe. Sci. Rep. 5:16951. 10.1038/srep16951 26581247 PMC4652271

[B11] Chen L.-Z. Kan Y. Zhang Z. Y. Wang Y.-L. Zhang X.-N. Wang X.-Y.. (2018). Neuropeptide initiated mast cell activation by transcutaneous electrical acupoint stimulation of acupoint LI4 in rats. Sci. Rep. 8:13921. 10.1038/s41598-018-32048-3 30224712 PMC6141543

[B12] Chen S. Shao L. Ma L. (2021). Cerebral edema formation after stroke: emphasis on blood-brain barrier and the lymphatic drainage system of the brain. Front. Cell. Neurosci. 15:716825. 10.3389/fncel.2021.716825 34483842 PMC8415457

[B13] Cheng C. Y. Lin J. G. Su S. Y. Tang N. Y. Kao S. T. Hsieh C. L.. (2014a). Electroacupuncture-like stimulation at Baihui and Dazhui acupoints exerts neuroprotective effects through activation of the brain-derived neurotrophic factor-mediated MEK1/2/ERK1/2/p90RSK/bad signaling pathway in mild transient focal cerebral ischemia in rats. BMC Compl. Altern. Med. 14:92. 10.1186/1472-6882-14-92 24606810 PMC3975570

[B14] Cheng C. Y. Lin J. G. Tang N. Y. Kao S. T. Hsieh C. L. (2014b). Electroacupuncture-like stimulation at the Baihui (GV20) and Dazhui (GV14) acupoints protects rats against subacute-phase cerebral ischemia-reperfusion injuries by reducing S100B-mediated neurotoxicity. PLoS ONE 9:e91426. 10.1371/journal.pone.0091426 24626220 PMC3953388

[B15] Cheng Y. Wang Y. J. (2020). Meningeal lymphatic vessels: a drain of the brain involved in neurodegeneration. Neurosci. Bull. 36, 557–560. 10.1007/s12264-019-00456-8 31893342 PMC7186281

[B16] Cho H. W. Hwang E. H. Lim B.. (2014). How current clinical practice guidelines for low back pain reflect traditional medicine in East Asian countries: a systematic review of clinical practice guidelines and systematic reviews. PLoS ONE 9:e88027. 10.1371/journal.pone.0088027 24505363 PMC3914865

[B17] Choi D. W. (1988). Glutamate neurotoxicity and diseases of the nervous system. Neuron 1, 623–634. 10.1016/0896-6273(88)90162-6 2908446

[B18] Choudhury G. R. Ding S. (2015). Reactive astrocytes and therapeutic potential in focal ischemic stroke. Neurobiol. Dis. (2016) 85, 234–244. 10.1016/j.nbd.2015.05.003 25982835 PMC4644522

[B19] Collmann F. M. Pijnenburg R. Hamzei-Taj S. Minassian A. Folz-Donahue K. Kukat C.. (2019). Individual *in vivo* profiles of microglia polarization after stroke, represented by the genes iNOS and Ym1. Front. Immunol. 10:1236. 10.3389/fimmu.2019.01236 31214190 PMC6558167

[B20] Colonna M. Butovsky O. (2017). Microglia function in the central nervous system during health and neurodegeneration. Annu. Rev. Immunol. 35, 441–468. 10.1146/annurev-immunol-051116-052358 28226226 PMC8167938

[B21] Dai M. (2016). Effect and mechanism of the metabolic pathways about acupuncture Neiguan on MCAO rat astrocytes glutamate (Master's thesis). Shandong University of Traditional Chinese Medicine. Available online at: https://kns.cnki.net/kcms2/article/abstract?v=wcPNn8Zia7NywCsfhMQnWLxyPgaEfNaDzBFNIQ-86Liz_WhccP76_867ycFdd73PpEktj-2Hqfq_4KhmUMXq54puyXjwnAB4HXAmZ45cMszd7UwEN-RZS70QmXlKtF-IYjOrCjmM61Mwo-94U7fgsw==uniplatform=NZKPTlanguage=CHS

[B22] Danbolt N. C. (2001). Glutamate uptake. Prog. Neurobiol. 65, 1–105. 10.1016/S0301-0082(00)00067-8 11369436

[B23] Datta A. Sarmah D. Mounica L. Kaur H. Kesharwani R. Verma G.. (2020). Cell death pathways in ischemic stroke and targeted pharmacotherapy. Transl. Stroke Res. 11, 1185–1202. 10.1007/s12975-020-00806-z 32219729

[B24] de Pablo Y. Nilsson M. Pekna M. Pekny M. (2013). Intermediate filaments are important for astrocyte response to oxidative stress induced by oxygen-glucose deprivation and reperfusion. Histochem. Cell Biol. 140, 81–91. 10.1007/s00418-013-1110-0 23756782

[B25] Ding N. Jiang J. Qin P. Wang Q. Hu J. (2018). Mast cells are important regulator of acupoint sensitization via the secretion of tryptase, 5-hydroxytryptamine, and histamine. PLoS ONE 13:e0194022. 10.1371/journal.pone.0194022 29513755 PMC5841809

[B26] Dobos G. J. Kirschbaum B. Choi K. E. (2012). The western model of integrative oncology: the contribution of Chinese medicine. Chin. J. Integr. Med. 18, 643–651. 10.1007/s11655-012-1200-1 22936317

[B27] Du Y. Shi L. Li J. Xiong J. Li B. Fan X.. (2011). Angiogenesis and improved cerebral blood flow in the ischemic boundary area were detected after electroacupuncture treatment to rats with ischemic stroke. Neurol. Res. 33, 101–107. 10.1179/016164110X12714125204317 20546685

[B28] Eming S. A. Krieg T. Davidson J. M. (2007). Inflammation in wound repair: molecular and cellular mechanisms. J. Invest. Dermatol. 127, 514–525. 10.1038/sj.jid.5700701 17299434

[B29] Feng D. Zhou J. Liu H. Wu X. Li F. Zhao J.. (2022). Astrocytic NDRG2-PPM1A interaction exacerbates blood-brain barrier disruption after subarachnoid hemorrhage. Sci. Adv. 8:eabq2423. 10.1126/sciadv.abq2423 36179025 PMC9524825

[B30] Franco-Bocanegra D. K. McAuley C. Nicoll J. Boche D. (2019). Molecular mechanisms of microglial motility: changes in ageing and Alzheimer's disease. Cells 8:639. 10.3390/cells8060639 31242692 PMC6627151

[B31] Gan Y. Liu Q. Wu W. Yin J.-X. Bai X-, F. Shen R.. (2014). Ischemic neurons recruit natural killer cells that accelerate brain infarction. Proc. Natl. Acad. Sci. U. S. A. 111, 2704–2709. 10.1073/pnas.1315943111 24550298 PMC3932858

[B32] Gelderblom M. Leypoldt F. Steinbach K. Behrens D. Choe C. U. Siler D. A.. (2009). Temporal and spatial dynamics of cerebral immune cell accumulation in stroke. Stroke 40, 1849–1857. 10.1161/STROKEAHA.108.534503 19265055

[B33] Gill D. Veltkamp R. (2016). Dynamics of T cell responses after stroke. Curr. Opin. Pharmacol. 26, 26–32. 10.1016/j.coph.2015.09.009 26452204

[B34] Han B. Lu Y. Zhao H. Wang Y. Li L. Wang T.. (2015). Electroacupuncture modulated the inflammatory reaction in MCAO rats via inhibiting the TLR4/NF-κB signaling pathway in microglia. Int. J. Clin. Exp. Pathol. 8, 11199–11205. 26617842 PMC4637657

[B35] Hoxhaj G. Manning B. D. (2020). The PI3K-AKT network at the interface of oncogenic signalling and cancer metabolism. Nat. Rev. Cancer. 20, 74–88. 10.1038/s41568-019-0216-7 31686003 PMC7314312

[B36] Hsieh C.-L. Chang Q.-Y. Lin I.-H, Lin, J.-G. Liu C.-H. Tang N.-Y.. (2006). The study of electroacupuncture on cerebral blood flow in rats with and without cerebral ischemia. Am. J. Chin. Med. 34, 351–361. 10.1142/S0192415X06003886 16552844

[B37] Iadecola C. Anrather J. (2011). Stroke research at a crossroad: asking the brain for directions. Nat. Neurosci. 14, 1363–1368. 10.1038/nn.2953 22030546 PMC3633153

[B38] Jelkmann W. (2005). Effects of erythropoietin on brain function. Curr. Pharm. Biotechnol. 6, 65–79. 10.2174/1389201053167257 15727557

[B39] Jeong H.-K. Ji K.-M. Min K.-J. Choi I. Choi D.-J. Jou I.. (2014). Astrogliosis is a possible player in preventing delayed neuronal death. Mol. Cells 37, 345–355. 10.14348/molcells.2014.0046 24802057 PMC4012084

[B40] Jia H. He J. Zhao L. Hsu C.-C. Zhao X. Du Y.. (2022). Combination of stem cell therapy and acupuncture to treat ischemic stroke: a prospective review. Stem Cell Res. Ther. 13:87. 10.1186/s13287-022-02761-y 35241146 PMC8896103

[B41] Jiang M. Liu X. Zhang D. Wang Y. Hu X. Xu F.. (2018). Celastrol treatment protects against acute ischemic stroke-induced brain injury by promoting an IL-33/ST2 axis-mediated microglia/macrophage M2 polarization. J. Neuroinflammation. 15:78. 10.1186/s12974-018-1124-6 29540209 PMC5853059

[B42] Jiang P. Chen C. Wang R. Chechneva O. V. Chung S. H. Rao M. S.. (2013). hESC-derived Olig2+ progenitors generate a subtype of astroglia with protective effects against ischaemic brain injury. Nat. Commun. 4:2196. 10.1038/ncomms3196 23880652 PMC3903179

[B43] Jin Q. Cheng J. Liu Y. Wu J. Wang X. Wei S.. (2014). Improvement of functional recovery by chronic metformin treatment is associated with enhanced alternative activation of microglia/macrophages and increased angiogenesis and neurogenesis following experimental stroke. Brain Behav. Immun. 40, 131–142. 10.1016/j.bbi.2014.03.003 24632338

[B44] Jin X. Jin L. Jin G. Y. (2019). The anti-inflammatory effect of acupuncture and its significance in analgesia. World J. Acupunct. Moxibust. 29, 1–6. 10.1016/j.wjam.2019.03.003

[B45] Jin X. L. (2017). Study on the effect of Electroacupuncture on antioxidative stress in mice with cerebral ischemia reperfusioninjury based on Nrf2 pathway (Master's thesis). Nanjing University of Traditional Chinese Medicine. 10.27253/d.cnki.gnjzu.2017.000004

[B46] Kim J. Kim N. Yenari M. A. (2015). Mechanisms and potential therapeutic applications of microglial activation after brain injury. CNS Neurosci. Ther. 21, 309–319. 10.1111/cns.12360 25475659 PMC4376565

[B47] Kim J.-T. Fonarow G. C. Smith E. E. Reeves M. J. Navalkele D. D. Grotta J. C.. (2017). Treatment with tissue plasminogen activator in the golden hour and the shape of the 4.5-hour time-benefit curve in the National United States get with the guidelines-stroke population. Circulation. 135, 128–139. 10.1161/CIRCULATIONAHA.116.023336 27815374

[B48] Kim J. H. Jung H. G. Kim A. Shim H. S. Hyeon S. J. Lee Y. S.. (2021). Hevin-calcyon interaction promotes synaptic reorganization after brain injury. Cell Death Differ. 28, 2571–2588. 10.1038/s41418-021-00772-5 33753902 PMC8408247

[B49] Kimura M. Tohya K. Kuroiwa K. Oda H. Gorawski E. C. Hua Z. X.. (1992). Electron microscopical and immunohistochemical studies on the induction of “Qi” employing needling manipulation. Am. J. Chin. Med. 20, 25–35. 10.1142/S0192415X92000047 1605128

[B50] Kobayashi M. Benakis C. Anderson C. Moore M. J. Poon C. Uekawa K.. (2019). AGO CLIP reveals an activated network for acute regulation of brain glutamate homeostasis in ischemic stroke. Cell Rep. 28, 979–991.e6. 10.1016/j.celrep.2019.06.075 31340158 PMC6784548

[B51] Lee M. J. Jang M. Choi J. Lee G. Min H. J. Chung W.-S.. (2016). Bee venom acupuncture alleviates experimental autoimmune encephalomyelitis by upregulating regulatory T cells and suppressing Th1 and Th17 responses. Mol. Neurobiol. 53, 1419–1445. 10.1007/s12035-014-9012-2 25579380

[B52] Li N. Liu T.-H. Yu J.-Z. Li C.-X. Liu Y. Wu Y.-Y.. (2019). Curcumin and curcumol inhibit NF-κB and TGF-β (1)/smads signaling pathways in CSE-treated RAW246.7 cells. Evid. Based Complement. Alternat. Med. 2019:3035125. 10.1155/2019/3035125 31007701 PMC6441512

[B53] Li P. Gan Y. Sun B. L. Zhang F. Lu B. Gao Y.. (2013). Adoptive regulatory T-cell therapy protects against cerebral ischemia. Ann. Neurol. 74, 458–471. 10.1002/ana.23815 23674483 PMC3748165

[B54] Li R. Liu W. Yin J. Chen Y. Guo S. Fan H.. (2018). TSG-6 attenuates inflammation-induced brain injury via modulation of microglial polarization in SAH rats through the SOCS3/STAT3 pathway. J. Neuroinflammation. 15:231. 10.1186/s12974-018-1279-1 30126439 PMC6102893

[B55] Li Y. Zhang J. Wan J. Liu A. Sun J. (2020). Melatonin regulates Abeta production/clearance balance and Abeta neurotoxicity: a potential therapeutic molecule for Alzheimer's disease. Biomed. Pharmacother. 132:110887. 10.1016/j.biopha.2020.110887 33254429

[B56] Li Z. Bi R. Sun S. Chen S. Chen J. Hu B.. (2022). The role of oxidative stress in acute ischemic stroke-related thrombosis. Oxid. Med. Cell. Longev. 2022:8418820. 10.1155/2022/8418820 36439687 PMC9683973

[B57] Liesz A. Suri-Payer E. Veltkamp C. Doerr H. Sommer C. Rivest S.. (2009). Regulatory T cells are key cerebroprotective immunomodulators in acute experimental stroke. Nat. Med. 15, 192–199. 10.1038/nm.1927 19169263

[B58] Lin R. Chen J. Li X. Mao J. Wu Y. Zhuo P.. (2016a). Electroacupuncture at the Baihui acupoint alleviates cognitive impairment and exerts neuroprotective effects by modulating the expression and processing of brain-derived neurotrophic factor in APP/PS1 transgenic mice. Mol. Med. Rep. 13, 1611–1617. 10.3892/mmr.2015.4751 26739187 PMC4732857

[B59] Lin R. Li X. Liu W. Chen W. Yu K. Zhao C.. (2017). Electro-acupuncture ameliorates cognitive impairment via improvement of brain-derived neurotropic factor-mediated hippocampal synaptic plasticity in cerebral ischemia-reperfusion injured rats. Exp. Ther. Med. 14, 2373–2379. 10.3892/etm.2017.4750 28962170 PMC5609168

[B60] Lin R. Lin Y. Tao J. Chen B. Yu K. Chen J.. (2015). Electroacupuncture ameliorates learning and memory in rats with cerebral ischemia-reperfusion injury by inhibiting oxidative stress and promoting p-CREB expression in the hippocampus. Mol. Med. Rep. 12, 6807–6814. 10.3892/mmr.2015.4321 26397995

[B61] Lin R. Wu Y. Tao J. Chen B. Chen J. Zhao C.. (2016b). Electroacupuncture improves cognitive function through Rho GTPases and enhances dendritic spine plasticity in rats with cerebral ischemia-reperfusion. Mol. Med. Rep. 13, 2655–2660. 10.3892/mmr.2016.4870 26846874

[B62] Lin X. Song F. Wu Y. Xue D. Wang Y (2022). Lycium barbarum polysaccharide attenuates Pseudomonas-aeruginosa pyocyanin-induced cellular injury in mice airway epithelial cells. Food Nutr. Res. 66. 10.29219/fnr.v66.4585 35261577 PMC8861857

[B63] Lipton S. A. Rosenberg P. A. (1994). Excitatory amino acids as a final common pathway for neurologic disorders. N. Engl. J. Med. 330, 613–622. 10.1056/NEJM199403033300907 7905600

[B64] Liu A. J. Li J. H. Li H. Q. Fu D. L. Lu L. Bian Z. X.. (2015). Electroacupuncture for acute ischemic stroke: a meta-analysis of randomized controlled trials. Am. J. Chin. Med. 43, 1541–1566. 10.1142/S0192415X15500883 26621442

[B65] Liu L. Q. Liu X. R. Zhao J. Y. Yan F. Wang R. L. Wen S. H.. (2018). Brain-selective mild hypothermia promotes long-term white matter integrity after ischemic stroke in mice. CNS Neurosci. Ther. 24, 1275–1285. 10.1111/cns.13061 30295998 PMC6489965

[B66] Liu R. Xu N. G. Yi W. (2002). Electroacupuncture attenuates inflammation after ischemic stroke by inhibiting NF-κB-mediated activation of microglia. Evid. Based Complement. Alternat. Med. 2020:8163052. 10.1155/2020/8163052 32922507 PMC7453260

[B67] Liu W. Wang X. Yang S. Huang J. Xue X. Zheng Y.. (2016a). Electroacupunctre improves motor impairment via inhibition of microglia-mediated neuroinflammation in the sensorimotor cortex after ischemic stroke. Life Sci. 151, 313–322. 10.1016/j.lfs.2016.01.045 26979777

[B68] Liu Y. Wang H. Wang X. Mu L. Kong Q. Wang D.. (2013). The mechanism of effective electroacupuncture on T cell response in rats with experimental autoimmune encephalomyelitis. PLoS ONE 8:e51573. 10.1371/journal.pone.0051573 23382807 PMC3557272

[B69] Liu Z. Yan S. Wu J. He L. Li N. Dong G.. (2016b). Acupuncture for chronic severe functional constipation: a randomized trial. Ann. Intern. Med. 165, 761–769. 10.7326/M15-3118 27618593

[B70] Lv T. Zhao B. Hu Q. Zhang X. (2021). the glymphatic system: a novel therapeutic target for stroke treatment. Front. Aging Neurosci. 13:689098. 10.3389/fnagi.2021.689098 34305569 PMC8297504

[B71] Lyu Z. Chan Y. Li Q. Zhang Q. Liu K. Xiang J.. (2021b). Destructive effects of pyroptosis on homeostasis of neuron survival associated with the dysfunctional BBB-glymphatic system and amyloid-beta accumulation after cerebral ischemia/reperfusion in rats. Neural Plas. 2021:4504363. 10.1155/2021/4504363 34434229 PMC8382555

[B72] Lyu Z. Li Q. Yu Z. Chan Y. Fu L. Li Y.. (2021a). Yi-Zhi-Fang-Dai formula exerts neuroprotective effects against pyroptosis and blood-brain barrier-glymphatic dysfunctions to prevent amyloid-beta acute accumulation after cerebral ischemia and reperfusion in rats. Front. Pharmacol. 12:791059. 10.3389/fphar.2021.791059 34975487 PMC8714930

[B73] Ma Y. Wang J. Wang Y. Yang G. Y. (2017). The biphasic function of microglia in ischemic stroke. Prog. Neurobiol. 157, 247–272. 10.1016/j.pneurobio.2016.01.005 26851161

[B74] MacPherson H. Richmond S. Bland M. Brealey S. Gabe R. Hopton A.. (2013). Acupuncture and counselling for depression in primary care: a randomised controlled trial. PLoS Med. 10:e1001518. 10.1371/journal.pmed.1001518 24086114 PMC3782410

[B75] Magnus T. Wiendl H. Kleinschnitz C. (2012). Immune mechanisms of stroke. Curr. Opin. Neurol. 25, 334–340. 10.1097/WCO.0b013e328352ede6 22547104

[B76] Meng X. L. Zhang D. L. Sui S. H. (2019). Acute remote ischemic preconditioning alleviates free radical injury and inflammatory response in cerebral ischemia/reperfusion rats. Exp. Ther. Med. 18, 1953–1960. 10.3892/etm.2019.7797 31410157 PMC6676222

[B77] Montaño A. Staff I. McCullough L. D. Fortunato G. (2013). Community implementation of intravenous thrombolysis for acute ischemic stroke in the 3- to 4.5-hour window. Am. J. Emerg. Med. (2013) 31, 1707–1709. 10.1016/j.ajem.2013.08.032 24060324

[B78] Moskowitz M. A. Lo E. H. Iadecola C. (2010). The science of stroke: mechanisms in search of treatments. Neuron. 67, 181–198. 10.1016/j.neuron.2010.07.002 20670828 PMC2957363

[B79] Neuhaus A. A. Couch Y. Hadley G. Buchan A. M. (2017). Neuroprotection in stroke: the importance of collaboration and reproducibility. Brain 140, 2079–2092. 10.1093/brain/awx126 28641383

[B80] Ornello R. Degan D. Tiseo C. Di Carmine C. Perciballi L. Pistoia F.. (2018). Distribution and temporal trends from 1993 to 2015 of ischemic stroke subtypes: a systematic review and meta-analysis. Stroke 49, 814–819. 10.1161/STROKEAHA.117.020031 29535272

[B81] Pajarillo E. Rizor A. Lee J. Aschner M. Lee E. (2019). The role of astrocytic glutamate transporters GLT-1 and GLAST in neurological disorders: potential targets for neurotherapeutics. Neuropharmacology. (2019) 161:107559. 10.1016/j.neuropharm.2019.03.002 30851309 PMC6731169

[B82] Pang J. Itano T. Sumitani K. Negi T. Miyamoto O. (2003). Electroacupuncture attenuates both glutamate release and hyperemia after transient ischemia in gerbils. Am. J. Chin. Med. 31, 295–303. 10.1142/S0192415X03000977 12856868

[B83] Park J. Park H.-H. Choi H. Kim Y. S. Yu H.-J. Lee K.-Y.. (2012). Coenzyme Q10 protects neural stem cells against hypoxia by enhancing survival signals. Brain Res. (2012) 1478:64–73. 10.1016/j.brainres.2012.08.025 23046589

[B84] Perez-de-Puig I. Miró-Mur F. Ferrer-Ferrer M. Gelpi E. Pedragosa J. Justicia C.. (2015). Neutrophil recruitment to the brain in mouse and human ischemic stroke. Acta Neuropathol. 129, 239–57. 10.1007/s00401-014-1381-0 25548073

[B85] Prass K. Meisel C. Höflich C. Braun J. Halle E. Wolf T.. (2003). Stroke-induced immunodeficiency promotes spontaneous bacterial infections and is mediated by sympathetic activation reversal by poststroke T helper cell type 1-like immunostimulation. J. Exp. Med. 198, 725–736. 10.1084/jem.20021098 12939340 PMC2194193

[B86] Qiu Y. (2013). Clinical observation on scalp acupuncture combined with rehabilitation training for hemiplegia after stroke. J. Acupunct. Tuina Sci. 11, 226–229. 10.1007/s11726-013-0696-z

[B87] Rasmussen M. K. Mestre H. Nedergaard M. (2018). The glymphatic pathway in neurological disorders. Lancet Neurol.17, 1016–1024. 10.1016/S1474-4422(18)30318-1 30353860 PMC6261373

[B88] Ren X. Liu S. Lian C. Li H. Li K. Li L.. (2021). Dysfunction of the glymphatic system as a potential mechanism of perioperative neurocognitive disorders. Front. Aging Neurosci. 13:659457. 10.3389/fnagi.2021.659457 34163349 PMC8215113

[B89] Roy Choudhury G. Ryou M. G. Poteet E. Wen Y. He R. Sun F.. (2014). Involvement of p38 MAPK in reactive astrogliosis induced by ischemic stroke. Brain Res. 1551, 45–58. 10.1016/j.brainres.2014.01.013 24440774 PMC3987968

[B90] Segawa K. Blumenthal Y. Yamawaki Y. Ohtsuki G. A. (2021). Destruction model of the vascular and lymphatic systems in the emergence of psychiatric symptoms. Biology 10:34. 10.3390/biology10010034 33419067 PMC7825436

[B91] Serra P. Amrani A. Yamanouchi J. Han B. Thiessen S. Utsugi T.. (2003). CD40 ligation releases immature dendritic cells from the control of regulatory CD4+CD25+ T cells. Immunity 19, 877–889. 10.1016/S1074-7613(03)00327-3 14670304

[B92] Shen M. H. Li Z. R. Xiang X. R. Niu W. M. (2009). Effect of electroacupuncture on cerebral cortex ultrastructure in rats with cerebral ischemia-reperfusion injury. Zhen Ci Yan Jiu. 34, 167–170. 19761109

[B93] Shen Y. Sun A. Wang Y. Cha D. Wang H. Wang F.. (2012). Upregulation of mesencephalic astrocyte-derived neurotrophic factor in glial cells is associated with ischemia-induced glial activation. J. Neuroinflamm. 9:254. 10.1186/1742-2094-9-254 23173607 PMC3576245

[B94] Sherafat A. Pfeiffer F. Reiss A. M. Wood W. M. Nishiyama A. (2021). Microglial neuropilin-1 promotes oligodendrocyte expansion during development and remyelination by trans-activating platelet-derived growth factor receptor. Nat. Commun. 12:2265. 10.1038/s41467-021-22532-2 33859199 PMC8050320

[B95] Shi L. Cao H.-M. Li Y. Xu S-, X. Zhang Y. Zhang Y.. (2017). Electroacupuncture improves neurovascular unit reconstruction by promoting collateral circulation and angiogenesis. Neural Regen Res. 12, 2000–2006. 10.4103/1673-5374.221156 29323038 PMC5784347

[B96] Shu Z. M. Shu X. D. Li H. Q. Sun Y. Shan H. Sun X. Y.. (2016). Ginkgolide B protects against ischemic stroke via modulating microglia polarization in mice. CNS Neurosci. Ther. 22, 729–739. 10.1111/cns.12577 27306494 PMC6492915

[B97] Souder D. C. Dreischmeier I. A. Smith A. B. Wright S. Martin S. A. Sagar M. A. K.. (2021). Rhesus monkeys as a translational model for late-onset Alzheimer's disease. Aging Cell 20:e13374. 10.1111/acel.13374 33951283 PMC8208787

[B98] Stelmashook E. V. Isaev N. K. Lozier E. R. Goryacheva E. S. Khaspekov L. G. (2011). Role of glutamine in neuronal survival and death during brain ischemia and hypoglycemia. Int. J. Neurosci. 121, 415–422. 10.3109/00207454.2011.570464 21574892

[B99] Sun L. Fan Y. Fan W. Sun J. Ai X. Qiao H.. (2020). Efficacy and safety of scalp acupuncture in improving neurological dysfunction after ischemic stroke: a protocol for systematic review and meta-analysis. Medicine 99:e21783. 10.1097/MD.0000000000021783 32846808 PMC7447452

[B100] Sun L. Zhang Y. Liu E. Ma Q. Anatol M. Han H.. (2019). The roles of astrocyte in the brain pathologies following ischemic stroke. Brain Inj. 33, 712–716. 10.1080/02699052.2018.1531311 30335519

[B101] Sun Y. Jiang J. Zhang Z. Yu P. Wang L. Xu C.. (2008). Antioxidative and thrombolytic TMP nitrone for treatment of ischemic stroke. Bioorg. Med. Chem. 16, 8868–8874. 10.1016/j.bmc.2008.08.075 18790647

[B102] Tanabe N. Kuboyama T. Tohda C. (2019). Matrine promotes neural circuit remodeling to regulate motor function in a mouse model of chronic spinal cord injury. Neural Regen. Res. 14, 1961–1967. 10.4103/1673-5374.259625 31290454 PMC6676875

[B103] Tao J. Zheng Y. Liu W. Yang S. Huang J. Xue X.. (2016). Electro-acupuncture at LI11 and ST36 acupoints exerts neuroprotective effects via reactive astrocyte proliferation after ischemia and reperfusion injury in rats. Brain Res. Bull. 120, 14–24. 10.1016/j.brainresbull.2015.10.011 26524137

[B104] Terada K. Murata A. Toki E. Goto S. Yamakawa H. Setoguchi S.. (2020). Atypical antipsychotic drug ziprasidone protects against rotenone-induced neurotoxicity: an *in vitro* study. Molecules 25:4206. 10.3390/molecules25184206 32937854 PMC7570562

[B105] Tian R. Mao G. (2022). Ghrelin reduces cerebral ischemic injury in rats by reducing M1 microglia/macrophages. Eur. J. Histochem. 66:3350. 10.4081/ejh.2022.3350 35016495 PMC8764466

[B106] Toro C. A. Zhang L. Cao J. Cai D. (2019). Sex differences in Alzheimer's disease: Understanding the molecular impact. Brain Res. 1719, 194–207. 10.1016/j.brainres.2019.05.031 31129153 PMC6750802

[B107] Vaitkiene P. Valiulyte I. Glebauskiene B. Liutkeviciene R. (2017). N-myc downstream-regulated gene 2 (NDRG2) promoter methylation and expression in pituitary adenoma. Diagn. Pathol. 12:33. 10.1186/s13000-017-0622-7 28390436 PMC5385074

[B108] Wang X. (2005). Investigational anti-inflammatory agents for the treatment of ischaemic brain injury. Expert Opin. Investig. Drugs. 14, 393–409. 10.1517/13543784.14.4.393 15882116

[B109] Wang X.-R. Shi G-, X. Yang J.-W. Yan C-, Q. Lin L.-T. Du S-, Q.. (2015). Acupuncture ameliorates cognitive impairment and hippocampus neuronal loss in experimental vascular dementia through Nrf2-mediated antioxidant response. Free Radic. Biol. Med. (2015) 89, 1077–1084. 10.1016/j.freeradbiomed.2015.10.426 26546103

[B110] Wei L. Chen C. Ding L. Mo M. Zou J. Lu Z.. (2019). Wnt1 promotes EAAT2 expression and mediates the protective effects of astrocytes on dopaminergic cells in Parkinson's disease. Neural Plast. 2019:1247276. 10.1155/2019/1247276 31582965 PMC6754970

[B111] World Health Organization (2019). WHO Global Report on Traditional and Complementary Medicine. Beijing: People's Medical Publishing House.

[B112] Wu X. D. Du L. N. Wu G. C. Cao X. D. (2001). Effects of electroacupuncture on blood-brain barrier after cerebral ischemia-reperfusion in rat. Acupunct. Electrother. Res. 26, 1–9. 10.3727/036012901816356063 11394489

[B113] Xin Z. Xue-Ting L. De-Ying K. (2015). GRADE in systematic reviews of acupuncture for stroke rehabilitation: recommendations based on high-quality evidence. Sci. Rep. 5:16582. 10.1038/srep16582 26560971 PMC4642304

[B114] Xu H. Zhang Y. Sun H. Chen S. Wang F. (2014). Effects of acupuncture at GV20 and ST36 on the expression of matrix metalloproteinase 2, aquaporin 4, and aquaporin 9 in rats subjected to cerebral ischemia/reperfusion injury. PLoS ONE 9:e97488. 10.1371/journal.pone.0097488 24828425 PMC4020847

[B115] Xu J. Yi M. Ding L. He S. A. (2019). Review of anti-inflammatory compounds from marine fungi, 2000-2018. Mar. Drugs. 17:636. 10.3390/md17110636 31717541 PMC6891400

[B116] Xu Y. Guo Y. Song Y. Zhang K. Zhang Y. Li Q.. (2018). A new theory for acupuncture: promoting robust regulation. J Acupunct Meridian Stud. 11, 39–43. 10.1016/j.jams.2017.11.004 29482800

[B117] Yang T. Sun Y. Zhang F. (2016). “The role of nonneuronal Nrf2 pathway in ischemic stroke: damage control and potential tissue repair,” in Non-Neuronal Mechanisms of Brain Damage and Repair After Stroke. Springer Series in Translational Stroke Research, eds Chen J. Zhang J. Hu X. (Cham: Springer).

[B118] Zhan J. Pan R. Zhou M. Tan F. Huang Z. Dong J.. (2018). Electroacupuncture as an adjunctive therapy for motor dysfunction in acute stroke survivors: a systematic review and meta-analyses. BMJ Open 8:e017153. 10.1136/bmjopen-2017-017153 29371267 PMC5786119

[B119] Zhang Q. Tian Z. X. (2020). Effects of Xingnao Kaiqiao acupuncture combined with Alteplase on neurological impairment, lipid peroxidation and cerebral vascular reserve function in patients with acute cerebral infarction. Shanghai J. Acupunct. 39, 25–30. 10.13460/j.issn.1005-0957.2020.01.0025

[B120] Zhang S. Jin T. Wang L. Liu W. Zhang Y. Zheng Y.. (2020). Electro-acupuncture promotes the differentiation of endogenous neural stem cells via exosomal microRNA 146b after ischemic stroke. Front. Cell. Neurosci. 14:223. 10.3389/fncel.2020.00223 32792909 PMC7385414

[B121] Zhao L. Chen J. Li Y. Sun X. Chang X. Zheng H.. (2017). The long-term effect of acupuncture for migraine prophylaxis: a randomized clinical trial. JAMA Intern. Med. 177, 508−515. 10.1001/jamainternmed.2016.9378 28241154

[B122] Zhao Q. Y. Ma X. D. (2018). Experimental study on the antioxidant effect of acupuncture Baihui and Sishencong on CI/RI model rats. J. Liaoning Univ. Trad. Chin. Med. 20, 66–69. 10.13194/j.issn.1673-842x.2018.08.018

[B123] Zhong X. Y. Ruan S. Wang F.. (2022). Mechanism of electroacupuncture alleviating cerebral ischemia reperfusion injury in rats by regulating endogenous melatonin secretion. Acupunct Res. 47, 39–49. 10.3724/SP.J.1329.2022.01006 35128869

[B124] Zhou X. Li Y. Lenahan C. Ou Y. Wang M. He Y.. (2021). Glymphatic system in the central nervous system, a novel therapeutic direction against brain edema after stroke. Front. Aging Neurosci. 13:698036. 10.3389/fnagi.2021.698036 34421575 PMC8372556

[B125] Zhu J. Cao D. Guo C. Liu M. Tao Y. Zhou J.. (2019). Berberine facilitates angiogenesis against ischemic stroke through modulating microglial polarization via AMPK signaling. Cell. Mol. Neurobiol. 39, 751–768. 10.1007/s10571-019-00675-7 31020571 PMC11462843

