# Supplementary material for: Mobilizing endogenous neuroprotection: the mechanism of the protective effect of acupuncture on the brain after stroke
Source: Front Neurosci. 2024 Apr 25;18:1181670. doi: 10.3389/fnins.2024.1181670 (PMC11084281; doi:10.3389/fnins.2024.1181670)
Supplement: Supplementary file 1 [file Table_1.DOCX]

**Appendix**

**TABLE 1丨Effect of acupuncture on microglia polarization after ischemic stroke**

| **Model/Objects** | **Intervention** | **Acupoints** | **Acupuncture parameters** | **Outcome** | **Biochemical measurements** | **Reference** |
| --- | --- | --- | --- | --- | --- | --- |
| permanent middle cerebral artery occlusion (pMCAO) rats | Electroacupuncture（EA） | GV 20、GV 14 | 20 times/min, 1-2 mA,  30 min once per day | Electroacupuncture inhibited the degeneration and necrosis of microglia in the cortical ischemic penumbra (IP) and ameliorated mitochondrial damage. | ①M1：Iba-1 ↓、CD11b ↓  ②NF-κB p65 ↓、IL-1β ↓、TNF -α ↓  ③the conversion of microglia to the M1 phenotype after ischemia ↓ | [96] Liu R, Xu NG, Yi W, Ji C. Electroacupuncture Attenuates Inflammation after Ischemic Stroke by Inhibiting NF-κB-Mediated Activation of Microglia. Evid Based Complement Alternat Med. 2020. 2020: 8163052. |
| middle cerebral artery occlusion and reperfusion (MCAO/R) rats | Electroacupuncture（EA） | LI 11、ST 36 | Dilatational wave of 1–20 Hz, peak voltage of 6 V, 0.2 mA,  30 min/day for 3days | EA treatment for MCAO rats showed a significant reduction in the infarct volumes accompanied by functional recovery in mNSS outcomes, motor function performances. | ①TNF-α,IL-1β and IL-6 ↓  ②the nucleus translocation of NF-κB p65 ↓  ③p38MAPK ↓、MyD88 ↓ | [97] Liu W, Wang X, Yang S, et al. Electroacupunctre improves motor impairment via inhibition of microglia-mediated neuroinflammation in the sensorimotor cortex after ischemic stroke. Life Sci. 2016. 151: 313-322. |
| middle cerebral artery occlusion (MCAO) rats | Electroacupuncture（EA） | LI 11、ST 36 | 4V, dense disperse wave of 1- or 20-Hz,  30 min once daily | EA alleviated the symptoms of neurological deficits and reduced the infarct volume in the rat brains. | ①miR-9 ↑  ②the expression of NF-κB signaling pathway-associated factors, NF-κB p65, TNF-α and IL-1β↓ | [98] Liu W, Wang X, Zheng Y, et al. Electroacupuncture inhibits inflammatory injury by targeting the miR-9-mediated NF-κB signaling pathway following ischemic stroke. Mol Med Rep. 2016. 13(2): 1618-26.  （Continued） |
| middle cerebral artery occlusion (MCAO) rats | Electroacupuncture（EA） | P 6、LI 11、SP 8 | the sparse-dense wave 2/15 Hz(The interval of the sparse wave and dense wave was 1.5 seconds.),1 mA,  30 min once per day for 5 days | Electroacupuncture can decrease the neurological score of MCAO model and the necrosis of hippocampal neuron and inhibit the aberrant expression of certain factors involved in the TLR4/NF-κB signaling pathway after cerebral ischemia. | ①the activation of the microglia ↓  ②TNF-α ↓、IL-1β ↓ 、IL-6 ↓ | [102] Han B, Lu Y, Zhao H, Wang Y, Li L, Wang T. Electroacupuncture modulated the inflammatory reaction in MCAO rats via inhibiting the TLR4/NF-κB signaling pathway in microglia. Int J Clin Exp Pathol. 2015. 8(9): 11199-205. |
| transient middle cerebral artery occlusion (tMCAO) rats | Electroacupuncture（EA） | GV20、LI 4、LR 3 | 1mA,2/20 Hz,  30min | EA treatment effectively improves ischaemic brain injury. EA treatment can promotes OTULIN gene transcription and protein synthesis in the brain, and enhanced microglial OTULIN expression. | ①endogenous OTULIN expression↑  ②the transformation of microglia and astrocytes from resting states to activated states ↓  ③NF-κB pathway↓ (IκBα phosphorylation and NF-κB p65 nuclear translocation ↑)  ④TNF-α, IL-1β and IL-6 ↓ | [103] Xu H, Wang Y, Luo Y. OTULIN is a new target of EA treatment in the alleviation of brain injury and glial cell activation via suppression of the NF-κB signalling pathway in acute ischaemic stroke rats. Mol Med. 2021. 27(1): 37. |
| middle cerebral artery occlusion (MCAO) rats | Electroacupuncture（EA）or α7nAChR agonist N-(3R)-1-azabicyclo[2.2.2]oct-3-yl-furo[2,3-c]pyridine-5-carboxamide hydrochloride (PHA-543,613 hydrochloride) and antagonist α-bungarotoxin (α-BGT) pretreatment. |  | 1 mA , 2/15 Hz ,  30 min per day for 5 days | ①EA pretreatment significantly reduced the infarction volume and improved the neurological deficit.  ②The activation of α7nAChR in microglia relieved the inflammatory response of primary microglia subjected to OGD and attenuated the injury of neurons co-cultured with microglia. | ①M1 markers (iNOS, IL-1β, and CD86) ↓  pro-inflammatory cytokines (TNF-α and IL-6) ↓  ②M2 markers (Arg-1, TGF-β1, and CD206) ↑  anti-inflammatory cytokines (IL-4 and IL-10) ↑ | [104] Ma Z, Zhang Z, Bai F, Jiang T, Yan C, Wang Q. Electroacupuncture Pretreatment Alleviates Cerebral Ischemic Injury Through α7 Nicotinic Acetylcholine Receptor-Mediated Phenotypic Conversion of Microglia. Front Cell Neurosci. 2019. 13: 537.  （Continued） |
| transient middle cerebral artery occlusion (tMCAO) rats | Electroacupuncture（EA） | GV 20、LI 4、LR 3 | 1 mA , 2/20 Hz ,  30 min once daily until the rats were sacrificed. | ①EA can induce microglial TREM2 expression in focal cerebral ischemia/reperfusion rats  ②EA treatment effectively promoted the phosphorylation of PI3K and AKT and suppressed the phosphorylation of NF-kB p65. | ①TNF-a, IL-1β and IL-6 ↓  ②IL-10 and Arg-1 ↑ | [105] Xu H, Mu S, Qin W. Microglia TREM2 is required for electroacupuncture to attenuate neuroinflammation in focal cerebral ischemia/reperfusion rats. Biochem Biophys Res Commun. 2018. 503(4): 3225-3234. |
| middle cerebral artery occlusion and reperfusion (MCAO/R) rats | Electroacupuncture（EA） | GV 20、LI 4、LR 3 | 1 mA, 20 Hz for 5min  and then 2 Hz for 30min | ①EA inhibited M1-like phenotypic microglial activation and promoted M2-like phenotypic microglia through upregulating CYLD expression.  ② EA-mediated modulation of the CX3CL1/CX3CR1 axis and NLRP3 inflammasome was reversed by CYLD gene silencing in the periischemic cortex. | ①neuronal CYLD expression ↑  ②M1 microglial ↓ , M2 microglial ↑ | [107] Lin X, Zhan J, Jiang J, Ren Y. Upregulation of Neuronal Cylindromatosis Expression is Essential for Electroacupuncture-Mediated Alleviation of Neuroinflammatory Injury by Regulating Microglial Polarization in Rats Subjected to Focal Cerebral Ischemia/Reperfusion. J Inflamm Res. 2021. 14: 2061-2078.  （Continued） |
| middle cerebral artery occlusion (MCAO) rats and middle cerebral artery occlusion and reperfusion (MCAO/R) rats | Electroacupuncture（EA） | GV20、ST36 | ①acupuncture groups :GV20、ST36 , twisted 180 degrees at a rate of 100±5 twists per min for 1 min, with the twisting procedure repeated at 10-min intervals for 20 min  ②electroacupuncture groups: GV20、ST36, continuous-wave 2 Hz (intensity, 1 mA) for 20 min | ①Acupuncture and electroacupuncture significantly decreased infarct size and improved neurological function.  ②electroacupuncture can significantly reduced target mRNA and protein levels and inflammatory cell. | ①the infiltration of inflammatory cells ↓  the expression of the proinflammatory enzyme, MMP2 ↓  ②the expression of the water channel proteins, AQP4 and AQP9↓ | [109] Xu H, Zhang Y, Sun H, Chen S, Wang F. Effects of acupuncture at GV20 and ST36 on the expression of matrix metalloproteinase 2, aquaporin 4, and aquaporin 9 in rats subjected to cerebral ischemia/reperfusion injury. PLoS One. 2014. 9(5): e97488. |
| middle cerebral artery occlusion and reperfusion (MCAO/R) rats | Electroacupuncture（EA） | DU 20、DU 24 | 0.2 mA, peak voltage of 6 V, and dilatational waves of 2/20 Hz,  30 min per day for 7 days | Astroglial and microglial/macrophage P2 purinoceptors-mediated neuroinflammation and hyperplasia in peri-infarct hippocampal CA1 and sensorimotor cortex were attenuated by EA treatment after ischemic stroke accompanied by the improved motor and memory behavior performance. | ①the level of pro-inflammatory cytokine interleukin-1β (IL-1β) ↓  ②the astroglial and microglial/macrophage P2 purinoceptors (P2X7R and P2Y1R)-mediated neuroinflammation ↓ | [111] Huang J, You X, Liu W, et al. Electroacupuncture ameliorating post-stroke cognitive impairments via inhibition of peri-infarct astroglial and microglial/macrophage P2 purinoceptors-mediated neuroinflammation and hyperplasia. BMC Complement Altern Med. 2017. 17(1): 480.  （Continued） |
